# The Impact of Employment Status and Children in Households on Food Security Among Syrian Refugees Residing in Florida

**DOI:** 10.7759/cureus.78751

**Published:** 2025-02-08

**Authors:** Racha Sankar, Catherine Coccia, Florance George, Fatma Huffman

**Affiliations:** 1 Dietetics and Nutrition, Florida International University, Miami, USA; 2 Health and Human Performance, Parker University, Dallas, USA; 3 Mathematics and Statistics, Florida International University, Miami, USA

**Keywords:** food security, household food security survey, refugees, syrian refugees, syrian war

## Abstract

Objectives: This study aims to measure food security and the levels of food insecurity among Syrian refugee households. It also aimed to determine the association between food security status and types of households including the number of employed members of the households and children in households.

Methods: Semi-structured interview questionnaires were administered to 80 households of Syrian refugees residing in Florida. Participants were Syrian refugees who have resettled in Florida since 2011 and were interviewed in one-on-one 45-minute sessions. Included cities were Miami, West Palm Beach, Orlando, and Tampa. The main outcomes were food security, levels of food insecurity, the number of employed individuals in households, and the structure of households with and without children.

Results: The mean food security score was 4.7± 2.6 among participating households when a score of 3-7 indicates food insecurity without hunger. There were significant differences (p = 0.02) between the levels of food insecurity in rural and urban areas. Households in rural areas experienced higher levels of food insecurity compared to households in urban areas. We found a significant relationship (p = 0.04) between food security and the number of employed individuals in households in rural areas. The logistic regression model comparing food security status in rural and urban areas showed that households in rural areas had 80.2 % less odds of being food secure than those in urban areas with the adjustment of the variable of number of employed individuals (odds ratio = 0.198; 95% CI: 0.055-0.712; p = 0.01). Another logistic regression model showed that Miami was four times and West Palm Beach was 11.8 times more likely to be food secure than Tampa when the number of employees was adjusted. Among all the households, there were significant differences (p = 0.01) in the levels of food insecurity between households with and without children. When the type of residence was introduced into the corresponding model, households in rural areas were 79.3% less likely to be food secure than households in urban areas (odds ratio: 0.207; CI: 0.06-0.70; p = 0.01). Another logistic regression showed that West Palm Beach had a significant positive effect (p = 0.005) on food security. Households in this city had 9.95 greater odds of being food secure than households in Tampa. The effect in Miami was marginally positive (p = 0.07) in this model. Households in Miami might have had 3.8 greater probabilities of being food secure than the households in Tampa when the variable of households with and without children was adjusted.

Conclusion: Food insecurity was frequent among n = 64 (80.0%) of Syrian refugee households residing in Florida. Households with at least two employed individuals were more likely to experience food security than households with only one member employed. The number of employees in households may have a greater impact on food security in urban areas than in rural areas. Food insecurity was more frequent in households with children than in households without children. Adults in food-insecure households with children might have experienced greater levels of food insecurity compared to their food-insecure children.

## Introduction

Food-insecure households are composed of individuals lacking sustainable access to sufficient food to maintain a healthy lifestyle [1]. Understanding the characteristics of households that may be associated with food security may help mitigate food insecurity. The income of households is one of the critical determinants of food insecurity and hunger in the United States (US) [2]. Among US households with children, more than one in five households lack maintainable access to nutritious food [3].

Employment status has a direct impact on the resources of households, which in turn affects food accessibility [4]. The US Bureau of Labor Statistics showed that an increase in the unemployment rate by 1% was associated with an increase of 0.5 percentage point in the prevalence of food insecurity between 2001 and 2012 [5]. At a household level, the result of these statistics showed that households headed by individuals holding a part-time job or with no labor hours were 12%-15% more likely to be food insecure than the households headed by full-time employees. The likelihood of food insecurity was 1.39% less in households with two employed individuals compared with households of one employed individual or households with no labor hours [5]. 

Employment status plays a key role in the economic security of households. Subsequently, economic security contributes to the differences in the level of food insecurity among US households with children [6]. A report by the United States Department of Agriculture (USDA) in 2000 showed that the prevalence of food insecurity was higher in households with children compared to the national average of food insecurity in the US [7]. This trend remained constant over a long period of time; the prevalence of food insecurity was 19% in households with children when it was 14% in all of the households in 2015 [8]. 

Several studies agreed that refugees living in the US were at an elevated risk of food insecurity [9-11]. Literature showed that there was an association between the number of employed individuals in households and food security in the US [4,5]. Households with children had a higher prevalence of food insecurity compared with the national average of food insecurity [8]. In accordance with records of the United Nations High Commissioner for Refugees (UNHCR), 75% of > 880,000 Syrian refugees fleeing the war were women and children [12]. Cultural norms of unemployed Syrian women fleeing with children may affect employment status, which in turn impacts the food security of Syrian refugees in the US. 

Accordingly, this work focused on measuring food security and the attributes of the structure of households including the number of employed individuals and the number of children on food security. The population of interest was Syrian refugees who resided in Florida after 2011. The differences in the levels of food insecurity between households with children and households without children were also tested. It is important to note that this research is part of a large dissertation project that aimed to investigate food security and the impact of various socioeconomic factors on food security among Syrian refugees residing in Florida [13]. 

## Materials and methods

The research model

A comprehensive model integrating food security with various socioeconomic factors was developed to guide our research. Since the effects of these factors on food security were assessed across different parts of the study, the model provided a consistent framework that focused solely on our variables of interest. Consequently, employment status, household types, and food access were identified as confounders influencing food security.

**Figure 1:**
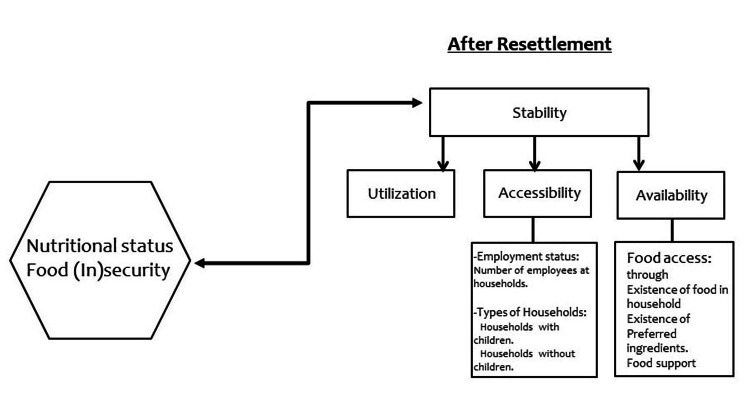
The model of the impact of employment status and types of households on food security among Syrian refugees in Florida

The model was structured around four constructs: utilization, accessibility, availability, and stability. Additionally, it included three key confounders: employment status, types of households, and food access. Utilization refers to the ability to purchase, prepare, and consume a balanced meal. Accessibility encompasses the resources available within social and physical environments. Availability pertains to the presence of resources. Stability represents the timeframe over which food security is sustainable. The employment status of family members and the presence of children in households were categorized under the Accessibility construct. The Food Security Model (FSM) developed by the USDA was our food security measurement tool (Appendix A) [14]; thus, we included variables such as the existence of food, preferred ingredients, and food support under the Availability construct (Figure 1). The development of our comprehensive model encompassing different socioeconomic factors, was detailed in a publication earlier in 2024 [15]. That publication provided an in-depth explanation of the model's core components and constructs. 

Design

The study lasted for five months and was conducted in the State of Florida. It started on August 1, 2019, and ended on November 19, 2019. Two semi-structured interview questionnaires that aimed to measure food security and to collect demographic characteristics of participants including the structure of households, number of employees, and number of children were administered to Syrian refugees living in Miami, West Palm Beach, Orlando, and Tampa. The questionnaires were translated from English to Arabic and then back-translated from Arabic to English by two Syrian immigrants fluent in both languages. A pilot study, involving six Syrian immigrants who voluntarily participated and arrived in the US after 2011, was conducted to validate the appropriateness of the questionnaires. The interviews were conducted in Arabic, the native language of the participants, and the interviewer. The approval of the Florida International University Institutional Review Board (FIU-IRB) was obtained as follows: the IRB protocol approval number is IRB-18-0301-CR01, and the TOPAZ reference number is 107023. English and Arabic versions of informed consent were developed and approved by FIU-IRB. 

Initially, the purpose of the research was communicated to the community leaders of the Syrian immigrant community in Miami. The Syrian immigrant community was responsible to assist newly arrived Syrian refugees in Miami. Thus, Syrian refugees were recruited with the assistance of the community leaders. Word of mouth was adopted eventually as another strategy to recruit our participants in Tampa, West Palm Beach, and Orlando. Tampa residents were mainly located in rural areas, while Miami, West Palm Beach, and Orlando were mainly urban dwellers.

The inclusion criteria were households of displaced Syrians in Florida who were originally registered by the United Nations (UN) as refugees and resettled in the United States after the beginning of the Syrian war in 2011. The exclusion criteria included Syrian immigrants who arrived in Florida before 2011, displaced Syrians who arrived in Florida after 2011 but were not registered by the UN, Syrian immigrants with different visa documentations, and asylum seekers with Syrian nationality residing in Florida. The recruitment process showed that there were 85 Syrian refugee households residing in Miami, Tampa, West Palm Beach, and Orlando. The purpose of the research was conveyed to all Syrian refugee households. Five households declined to participate when 80 households agreed to be interviewed.

Semi-structured Interview Questionnaires

Food security, levels of food insecurity, the employment status of members of households, and the number of children were our measurable outcomes. Multiple questionnaires were compiled into a comprehensive questionnaire with the objective of measuring food security and nutrition knowledge. As part of the demographic characteristics, information on gender, type of households, number of children, and employment status were collected (Appendix B). In one-on-one sessions, the comprehensive questionnaire was completed with an average of 45 minutes per session. Interestingly, such questionnaires triggered further explanations from interviewees; comments and information obtained were documented for future qualitative analysis. We also obtained data on other variables that were involved in our model to be examined and analyzed based on conclusions drawn from the comprehensive literature review.

Food Security

The FSM by the USDA was adopted to measure food security (Appendix A) [14]. Child-related questions were omitted for households without children. A total score of 10 points was given to such households, and a total score of 16 was given to households with children. Food insecurity was classified into three levels of severity; the greater number of affirmative responses indicated greater severity of food insecurity. Appendix C presented the FSM-USDA model, the levels of food insecurity, assessment questions, and the scoring system.

Employment Status and Types of Households

A series of questions were asked to obtain information about the number of family members earning income from employment, status of employment, main income earner, second main income earner, and other financial resources. The demographic section included questions about children, the number of children, gender, and age.

Statistical analysis

SAS Studio University Edition was used for all statistical analyses. For descriptive statistics, the one-way frequency was used to identify Syrian refugees in regard to demographic characteristics and variables of interest. The chi-square test was utilized to determine the differences in employment status in different types of residence. Fisher’s exact test and logistic regression were used to examine the association between food security and the characteristics of households in terms of employment status. These statistical tests were run to determine the association between the levels of food insecurity and food security status in households with and without children.

## Results

Table 1 demonstrated selected demographic characteristics of our participants including gender of respondents, employment status in households, and types of households.

**Table 1:** Demonstration of selected demographic characteristics of the participants including gender, employment status, and types of households (%) Column-based percentages within a specific category

Characteristic	n (%)
Gender of respondents	
Female	63 (78.7)
Male	17 (21.3)
Employment status in households	
Households with one employed individual	65 (81.3)
Households with two employed individuals	15 (18.7)
Types of households	
Households with children	71 (88.7)
Households without children	9 (11.3)

Food security

Of the 80 households, 20% were food secure, while 80% of households experienced food insecurity at different levels, the mean food security score was 4.7 ± 2.6. According to the FSM-USDA score, households of Syrian refugees in rural areas (n = 43) were moderately food insecure with hunger (5.00 ± 2.4), and households of Syrian refugees in urban areas (n = 37) were food insecure without hunger (4.50 ± 2.8). Fisher’s exact test showed that there were significant differences between the levels of food insecurity in rural and urban areas (p = 0.02) (Table 2). It also showed significant differences in the levels of food insecurity in different cities (p = 0.04) (Table 3).

**Table 2:** Levels of food insecurity and employment status by types of residence **Fisher’s exact test; statistically significant, p = <0.05; column-based percentages within a specific category

Characteristic	Rural areas n (%)	Urban areas n(%)	p-value
Number of respondents	43	37	
Level of food insecurity			0.02**
Food security	4 (9.3)	12 (32.4)	
Food insecurity without hunger	31 (72.1)	18 (48.6)	
Moderate food insecurity with hunger	8 (18.6)	5 (13.5)	
Severe food insecurity with hunger	0	2 (5.5)	

**Table 3:** Levels of food insecurity by city of residence **Fisher’s exact test; statistically significant, p = <0.05; column-based percentages within specific category

Level of food insecurity	Miami n(%)	West Palm Beach n(%)	Orlando n(%)	Tampa n(%)	p-value
Number of respondents	18 (100.0)	10 (100.0)	9 (100.0)	43 (100.0)	
Level of food insecurity					0.04**
Food security	5 (27.8)	5 (50.0)	2 (22.2)	4 (9.3)	
Food insecurity without hunger	9 (50.0)	3 (30.0)	6 (66.7)	31 (72.1)	
Moderate food insecurity with hunger	3 (16.7)	1 (10.0)	1 (11.1)	8 (18.6)	
Severe food insecurity with hunger	1 (5.5)	1 (10.0)	0	0	

Employment status

Table 4 demonstrates the employment status of participating households in urban and rural areas. The chi-square test showed that the difference in employment status was marginally significant in urban and rural areas (p = 0.09) (Table 4).

**Table 4:** Employment status by types of residence *Chi-square test; statistically significant, p = <0.05; column-based percentages within specific category

Characteristic	Rural areas n (%)	Urban areas n(%)	p-value
Number of respondents	43	37	
Employment status			0.09*
Households with one employed individual	32 (74.4)	33 (89.2)	
Households with two employed individuals	11 (25.6)	4 (10.8)	

Data collected lacked food-secure households with two employed individuals in urban areas including Miami, West Palm Beach, and Orlando. Nevertheless, food insecurity was frequent in n = 25 (64.1%) of all households in urban areas. Of these households, there were n = 21 (84.0%) households with one employed individual and n = 4 (16.0%) households with two employed individuals. In rural areas, Tampa food-insecure households with one employed individual accounted for 72% of all Syrian refugee households. Food-insecure households with two employed individuals accounted for 18.6%. The percentage of food security in households with one employee was 2.4%, whereas it was 7.0% in households with two employees in Tampa. Fisher’s exact test showed that there was a significant relation between food security status and the number of employed individuals in households in Tampa (p = 0.04) when there were no significant relations between food security status and the number of employed individuals in households in other cities (Table 5).

**Table 5:** Food security and employment status of households by types of residence and city of residence **Fisher’s exact test; statistically significant, p = <0.05; ^+^column-based percentages within specific category

Variables	Number of respondents (n)	Food-secure households n(%)	Food-insecure households n(%)	p-value
Rural areas (Tampa)	43			0.04**
Households with one employed individual		1 (25.0)	31 (79.5)	
Households with two employed individuals		3 (75.0)	8 (20.5)	
Urban areas (Miami, Orlando, West Palm Beach)	37			0.3**
Households with one employed individual		12 (100.0)	21 (84.0)	
Households with two employed individuals		0	4 (16.0)	
Miami	18			0.5**
Households with one employed individual		5 (100.0)	10 (76.9)	
Households with two employed individuals		0	3 (23.1)	
Orlando	9			1.0**
Households with one employed individual		2 (100.0)	6 (85.7)	
Households with two employed individuals		0	1 (14.3)	
West Palm Beach	10			
Households with one employed individual		5 (100.0)	5 (100.0)	
Households with two employed individuals		0	0	

The results of our logistic regression model showed that households with one employed individual did not have a significant effect on food insecurity compared to households with two employed individuals (Model 1, Table 6). When the variable of types of residence was added to the model (Model 2, Table 6), the model showed that households in rural areas had 80.2% less probability of being food secure than those in urban areas (odds ratio = 0.198; 95% CI: 0.055-0.712; p = 0.01) (Table 6).

**Table 6:** Logistic regression model demonstrating the effect of employment status of households on food security status in rural and urban areas (-) Reference group; β: estimate; B: odds ratio; SE: standard error; statistically significant, p < 0.05

Model terms	Model 1				Model 2			
Covariate	β	B	SE	p-value	β	B	SE	p-value
Constant	-1.4		0.64	0.03	-0.4		0.8	0.6
Employment status in households								
Households with one employed individual	0	1	0.7	1.0		0.65	0.8	0.6
Households with two employed individuals	-	-	-			-	-	
Types of residence								
Rural areas					-1.6	0.198	0.7	0.01
Urban areas						-	-	

Another logistic regression model (Model 3, Table 7), in which we adjusted the number of employed individuals, showed that Miami City had a marginally significant effect on food security (p = 0.06). The odds of being food secure were four times higher in Miami than in Tampa. The effect of West Palm Beach was significant (p = 0.004); the odds of being food secure for households in West Palm Beach were 11.8 times greater than for the households in Tampa.

**Table 7:** Logistic regression model demonstrating the effect of employment status of households on food security status in city of residence (-) Reference group; β: estimate; B: odds ratio; SE: standard error; statistically significant, p < 0.05

Model terms	Model 1				Model 3			
Covariate	β	B	SE	p-value	β	B	SE	p-value
Constant	-1.4		0.64	0.03	-1.8		0.7	0.01
Employment status in households								
Households with one employed individual	0	1	0.7	1.0	-0.6	0.54	0.8	0.4
Households with two employed individuals	-	-	-			-	-	
City of residence								
Miami					1.4	4.0	0.8	0.06
Orlando					1.1	3.11	1.0	0.24
West Palm Beach					2.5	11.8	0.9	0.004
Tampa						-	-	

Types of households

The frequency of food security was n = 14 (19.7%) among households with children, and two levels of food insecurity were observed among these households. The level of food insecurity without hunger was frequent in n = 44 (62.0%), and the level of moderate food insecurity with hunger was frequent among n = 13 (18.3%) of households with children. 

Table 8 demonstrates the levels of food insecurity in households with and without children in rural and urban areas. It also included levels of food insecurity in all cities. Of households without children, the frequency of food security was n = 2 (22.2%), while the frequency of food insecurity without hunger and severe food insecurity with hunger were n = 5 (55.6%) and n = 2 (22.2%), respectively. Thus, households with children did not experience severe levels of food insecurity, while households without children did not experience moderate levels of food insecurity with hunger. The result of Fisher’s exact test showed that there were significant differences in the levels of food insecurity between households with children and households without children in the entire population (p = 0.01) (Table 8). 

When testing such an association by the type of residence, the result showed that there were also significant differences in the levels of food insecurity between households with children and households without children in urban areas (p = 0.004) (Table 8). Fisher’s exact test was carried out again to determine the cities in which differences in the levels of food insecurity between households with and without children would be detected. The result showed that such differences were marginal in Miami (p = 0.07), and there were no significant differences in West Palm Beach (p = 0.2) (Table 8). Orlando and Tampa were excluded in this comparison; Orlando City did not have households without children; and Tampa City did not have severely food-insecure households with and without children.

**Table 8:** Levels of food insecurity in households with and without children by types of residence and city of residence **Fisher’s exact test; statistically significant, p = <0.05;^+^column-based percentages within specific category

Variables	Number of responders (n)	Food security n(%)	Food insecurity without hunger n(%)	Moderate food insecurity with hunger n(%)	Severe food insecurity with hunger n(%)	p-value
All households	80					0.01**
Households without children	9	2 (12.5)	5 (10.2)	0	2 (100.0)	
Households with children	71	14 (87.5)	44 (89.8)	13 (100.0)	0	
Rural areas (Tampa)	43					
Households without children		1 (25.0)	5 (16.1)	0	0	
Households with children		3 (75.0)	26 (83.9)	8 (100.0)	0	
Urban areas (Miami, West Palm Beach, Orlando)	37					0.004**
Households without children		1 (8.3)	0	0	2 (100.0)	
Households with children		11 (91.7)	18 (100.0)	5 (100.0)	0	
Miami	18					0.07**
Households without children		1 (20.0)	0	0	1 (100.0)	
Households with children		4 (80.0)	9 (100.0)	3 (100.0)	0	
West Palm Beach	10					0.2**
Households without children		0	0	0	1 (100.0)	
Households with children		5 (50.0)	3 (100.0)	1 (100.0)	0	

The FSM-USDA revealed that 83.1% of Syrian refugee children consumed low-cost food, and 70.4% were not constantly fed balanced meals in the past 12 months. Of the total households, 23.9% reduced portions of meals to children, and 7% of children had to skip meals sometimes throughout the past year. Although our findings showed that some households were food insecure at the hunger level, none of the households reported an event of hungry children without sufficient resources to buy food or that a child spent a whole day without food intake (Table 9).

**Table 9:** Percentage of affirmative responses to each item of FSM-USDA within the last 12 months FSM-USDA: Food Security Model-United States Department of Agriculture

Concerns for food availability	Percentage of affirmative response (always true/sometimes true)
Worried food would run out	51.3%
Food bought did not last	80.0%
Could not afford to eat balanced meals	77.5%
Few kinds of low-cost food for children	83.1%
Could not feed children a balanced meal	70.4%
Children were not eating enough	15.5%
Adults cut or skipped meals	20.0%
Respondent ate less than felt they should have	25.0%
Respondent was hungry, no resources to buy food	3.8%
Respondent lost weight loss due to lack of food	2.5%
An adult spent a whole day without food intake	5.0%
Reduced portions of meals to children	23.9%
Children did skip a meal	7.0%
Children were hungry, no resources to buy food	0
A child spent a whole day without eating	0

Our logistic regression did not show an effect of households with and without children on food security (Model 1, Table 10). When the variable of types of residence was incorporated into the model (Model 2, Table 10); there was a significant negative effect in rural areas (p = 0.01). Households in rural areas were 79.3% less likely to be food secure compared with households in urban areas when controlling households with and without children (odds ratio: 0.207; CI: 0.06-0.70; p = 0.01). 

**Table 10:** Logistic regression model demonstrating the effect of children in households on food security status in rural and urban areas (-) Reference group; β: estimate; B: odds ratio; SE: standard error; statistically significant, p < 0.05

Model Terms	Model 1				Model 2			
Covariate	β	B	SE	P value	β	B	SE	P value
Constant	-1.4		0.3	<0.0001	-0.8		0.4	0.03
Types of households								
Households without children	0.15	1.2	0.8	0.9	0.4	1.5	0.9	0.6
Households with children	-	-	-		-	-	-	
Types of residence								
Rural areas					-1.6	0.207	0.6	0.01
Urban areas					-	-	-	

Additionally, another logistic regression whose variables were cities of residence (Model 3, Table 11), controlling households with and without children, showed that West Palm Beach had a significant positive effect on food security (p = 0.005). The odds of being food secure were 9.9 times greater for households in West Palm Beach compared with households in Tampa City. The effect in Miami City was marginally positive (p = 0.07). The odds of being food secure could have been 3.8 times greater in Miami compared with households in Tampa. 

**Table 11:** Logistic regression model demonstrating the effect of children in households on food security status in city of residence (-) Reference group; β: estimate; B: odds ratio; SE: standard error; statistically significant, p < 0.05

Model terms	Model 1				Model 3			
Covariate	β	B	SE	p-value	β	B	SE	p-value
Constant	-1.3		0.3	<0.0001	-2.3		0.55	<0.0001
Types of households								
Households without children	-0.6	0.54	1.1	0.6	0.4	1.4	0.9	0.7
Households with children	-	-	-		-	-	-	
City of residence								
Miami					1.3	3.8	0.7	0.07
West Palm Beach					2.3	9.9	0.8	0.005
Orlando					1.1	2.9	1.0	0.3
Tampa					-	-	-	

## Discussion

Food insecurity is usually experienced by refugees residing in the United States (US). In the US northeast region, food insecurity was frequent among 85% of refugees [16]. Similarly, we found that food insecurity was frequent among 64(80%) of Syrian refugees residing in Florida. Among these food-insecure households, 39 (60.9%) were households in rural areas, and 25(39.1%) were households in urban areas. According to a US Economic Research report, US rural areas are prone to food insecurity compared with urban areas [17]. This report showed that the prevalence of food insecurity was 15.4% and 14.1% in rural and urban areas, respectively [17].

Besides the types of residence, the characteristics of households had different effects on the food security status and the levels of food insecurity in our population. In Tampa, as in the rural areas in our research, food insecurity was more frequent in households with one employed individual than in households with two employed individuals, 72.0% versus 18.6%. In addition, the levels of food insecurity were significantly different in this area.

Our logistic regression model (Model 2, Table 6) revealed that Tampa had fewer probabilities of being food secure than urban areas when the number of employed individuals was adjusted. Another logistic regression model (Model 3, Table 7) showed that adjustment of the number of employed individuals had a marginally significant effect on food security in Miami. The likelihood of being food secure could have been 300% greater in Miami than in Tampa. The probability of food security was 11.8 times greater in West Palm Beach compared with Tampa.

Therefore, our hypothesis of “households that have at least two employed family members are less likely to be food insecure” was supported. Additionally, the number of employed individuals per household may have a greater impact on food security in urban areas than in rural areas in our population. Food security was associated with the number of employed individuals in households in Tampa. When the number of employed individuals in households was adjusted, households in Miami and West Palm Beach had greater chances of being food secure compared with households in Tampa.

In fact, our rationale corresponded with the international concept of food security; food insecurity is associated with purchase power and food affordability in urban areas, and it is associated with the availability of food in rural areas [18]. The cost of living might have been an additional barrier to food security among Syrian refugees in urban areas resulting in marginal food budgets, which would have been expanded by additional employed individuals in households. Full-time employment was associated with a reduction in the affirmative responses on the food security scale by 1.3 points [19]. 

A study found that the employment status in households affected the stability of households’ income, and the greater the change in income was associated with a greater change in the severity of food insecurity [19]. Another study conducted in the US found that the cost of living of households was significantly higher in urban areas than in rural areas. Households in rural areas tended to spend a greater percentage of income on food compared with households in urban areas, 19.08% versus 15.56% [20]. Among US households, food-secure households spent 23% more on food than food-insecure households [21]. 

The other confounder, the types of households, had an effect on the construct of accessibility in our model (Figure 1). Households with and without children were utilized to determine the impact of such structures of households on food security among Syrian refugees in Florida. The majority of our population n = 71 (88.75%) were of households with children; 75% of Syrian refugees registered by the UN were women and children [12]. We found significant differences in the levels of food insecurity in households with and without children. Food insecurity was more frequent in Syrian refugee households with children than counterpart households without children, n = 57 (80.3%) versus n = 7 (77.8%). Food insecurity was prevalent among 11.8% of all US households and 15.7% of US households with children, as per the USDA 2018 annual report [21]. 

We hypothesized that households with children are more likely to be food insecure compared to households without children. Since we were able to detect significant differences in the levels of food insecurity among households with and without children, we further investigated our hypothesis. Our regression model (Model 2, Table 10) showed that households in rural areas were 79.3% less likely to be food secure than households in urban areas when the variable of households with and without children was adjusted. 

We carried out another logistic regression (Model 3, Table 11) that also confirmed that households in urban cities were more likely to be food secure compared with those households in rural areas when the same variable was adjusted. West Palm Beach was 9.9 times more likely to be food secure than Tampa. A marginal significant effect on food security was detected in Miami, and the odds of being food secure could have been 3.8 times greater in Miami than in Tampa. Our small sample size might have been a reason for the marginal effect in Miami. The inability to run the statistical test for Orlando was due to a lack of households without children in this city.

When analyzing the items in the FSM-USDA (Table 8), there was no household with children who did not eat an entire day, but 5.0% of households had an affirmative response to having an adult spending an entire day without eating due to lack of financial resources to purchase food. Hunger, as an acute feeling, was never experienced by children in the participating households, but it was experienced by 3.8% of adults in households participating in this research. The proportion of households with children who skipped meals was 7.0%, but the percentage of households with adults who skipped meals was 20.0%. Thus, meals were not equally distributed among members of Syrian refugee households, and their children were given preference to be fed. The price of food was a concern in 77.5% of all Syrian refugee households and 70.4% of Syrian refugee households with children since these households were unable to obtain balanced meals consistently. This concern was also indicated by 83.1% of households that had to purchase low-cost foods for children as a coping strategy. A study found that the number of food-insecure households with children was greater than the number of food-insecure children among the same population. Adults of such households reduced their intake to cope with food shortages and limited financial resources [22]. 

Therefore, Syrian households with children were more likely to be food insecure than Syrian refugee households without children. The likelihood of being food-insecure households with children was greater in rural areas than in urban areas. Adults in households with children faced difficulties feeding children a nutritionally adequate diet; adults tended to sacrifice to mitigate hunger among children.

Translation of our findings into the developed model

Initially, our developed model suggested that employment status, as a confounder, would be an indicator for food security. Our result confirmed that employment status under the accessibility construct had a direct effect on the stability construct resulting in changes in food security status between households with different numbers of employed individuals.

The variable of households with children was the second confounder that we listed under the accessibility construct. Our findings confirmed that children in households had an effect on the accessibility construct leading to a change in the stability construct. A new link between the constructs of accessibility and availability was also detected in this research. Households with children would have an effect on the availability construct among adults.

The focused assessment of food security among Syrian refugees in Florida was a key strength of this research. Our results align with previously published findings that explored the impact of employment status and structures of households on food security among various refugee populations residing in the US. Our results add compelling evidence that may provide a foundational source for developing appropriate interventions for Syrian refugees in future research. The researcher’s Syrian background along with the familiarity with Syrian culture and traditions greatly facilitated the recruitment process and data collection.

The sample size of this research was our main limitation. There were no households without children in Orlando City, while Tampa did not have severely food-insecure households with and without children. This prevented us from including Orlando in any corresponding statistical tests, and we were unable to include both cities when examining the association between the structure of households and the levels of food insecurity in urban and rural areas. Nevertheless, the power analysis showed that the number of participating households was sufficient. We had hoped to detect more significant findings to inform future intervention research. A larger sample size would provide stronger evidence to tailor remedial actions in urban and rural settings.

## Conclusions

Food insecurity was frequent among the majority of Syrian refugee households residing in Florida. Although households of Syrian refugees in urban and rural areas scored moderately food secure when applying the FSM-USDA, households in rural areas had greater food insecurity scores than expected, which reached hunger levels of “always” or “sometimes” on the Likert scale in the past 12 months.

The number of employed individuals in households and households with and without children were two determinants of food security among our population. Households with more than one employee were more likely to experience food security than households with one employed individual. In rural areas, Syrian refugees with households of two employed individuals might have experienced food insecurity due to a lack of physical availability of food. The high cost of living in urban areas might have created an indirect challenge to achieve food security; such a challenge could have been combated by an additional financial resource, the income of an employed family member.

Households with children tended to be more food insecure than households without children. Households with children in rural areas were at higher risk for food insecurity than households with children in urban areas. The levels of food insecurity might have varied among members of households with children; adults might have experienced greater food insecurity than children.

Regarding our food security model, we concluded that households with children might be considered a confounding variable affecting the construct of accessibility of food among all family members. It is also a potential confounder that might affect the construct of availability among adults. Further research might be needed to determine whether members of food-insecure households with children experience different levels of food insecurity among Syrian refugees.

## Figures and Tables

**Figure 2:**
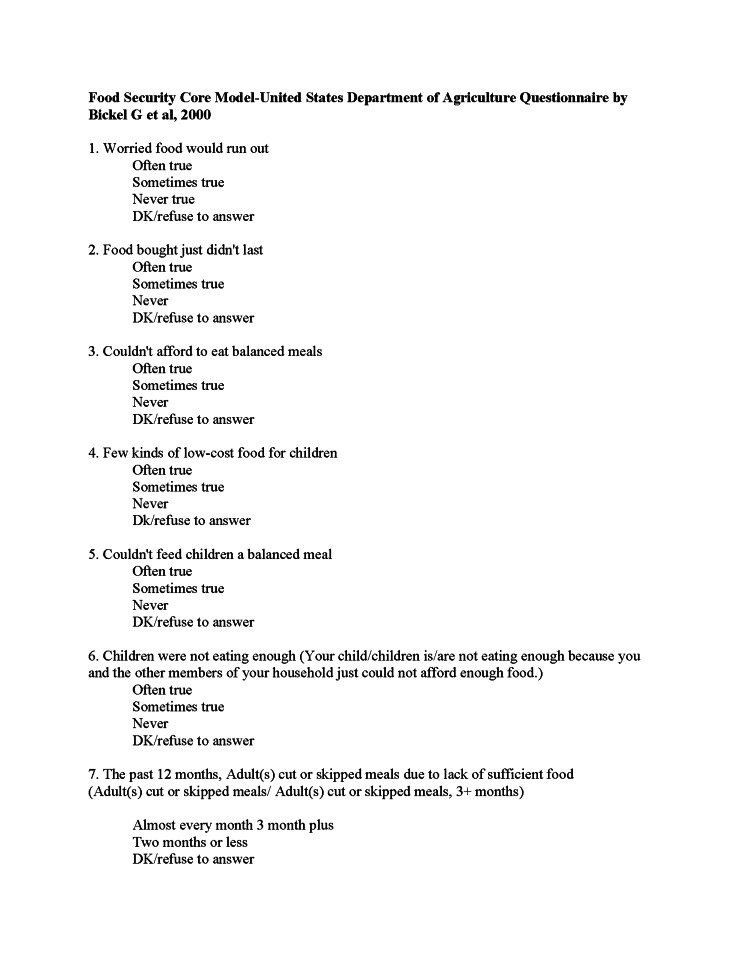
FSM-USDA: Food Security Core Model-United States Department of Agriculture

**Figure 3:**
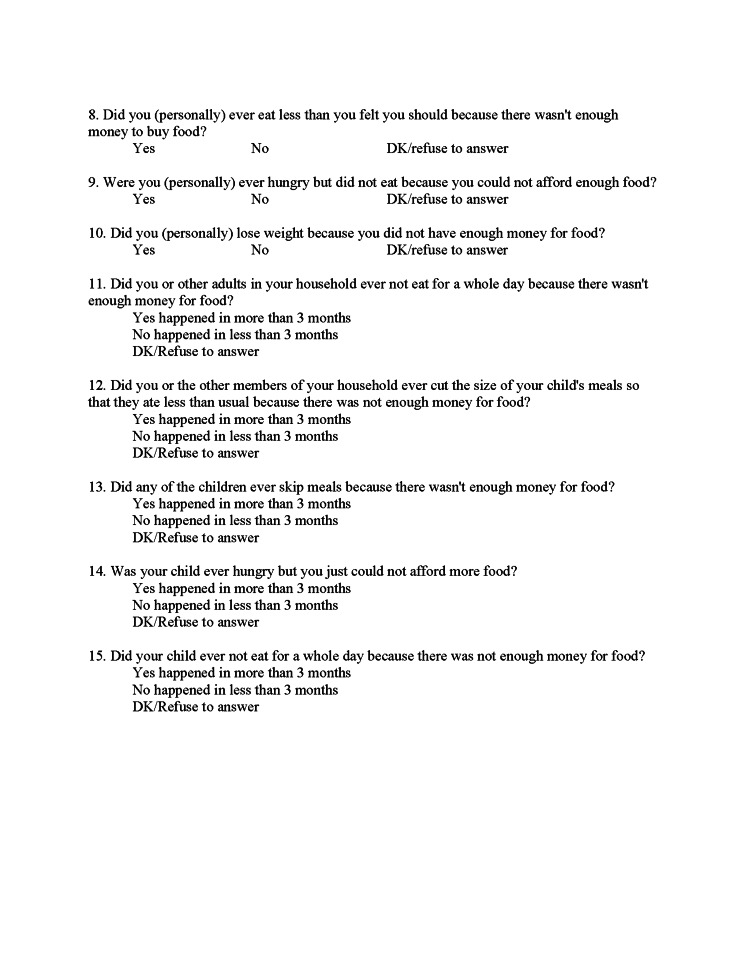
FSM-USDA: Food Security Core Model-United States Department of Agriculture (Contd.)

**Figure 4:**
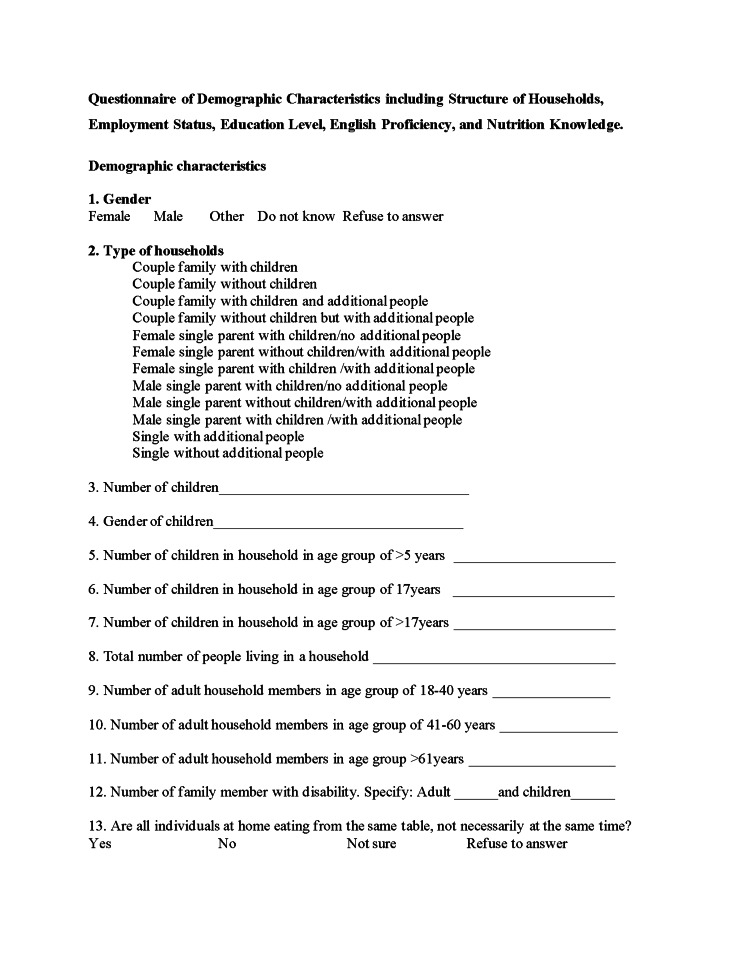
The Comprehensive Questionnaire

**Figure 5:**
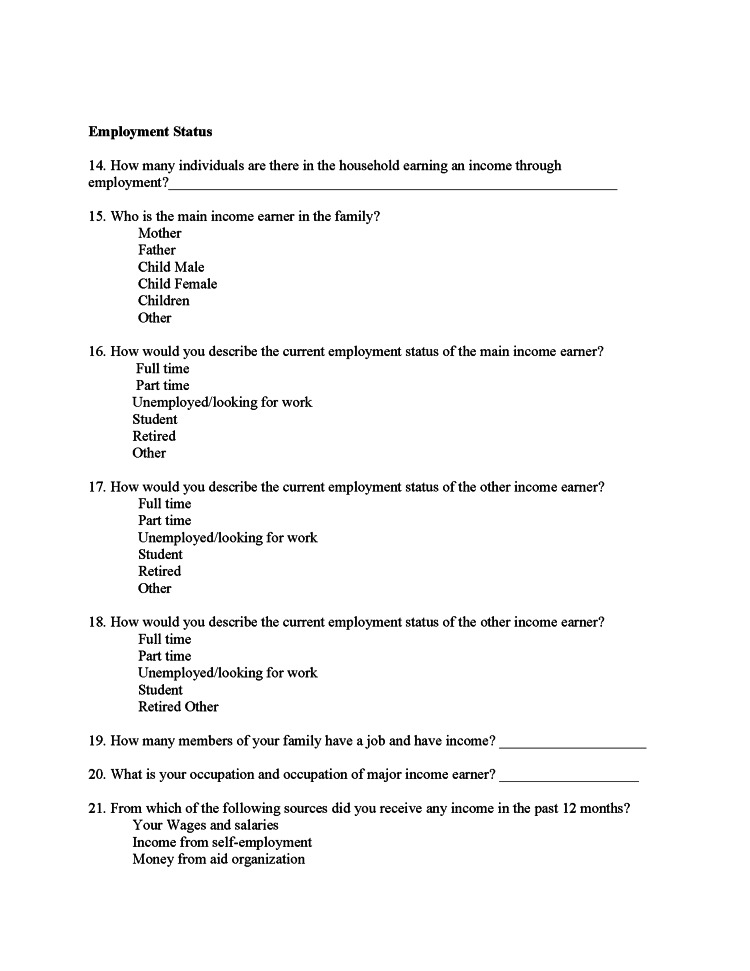
The Comprehensive Questionnaire (Contd.)

**Figure 6:**
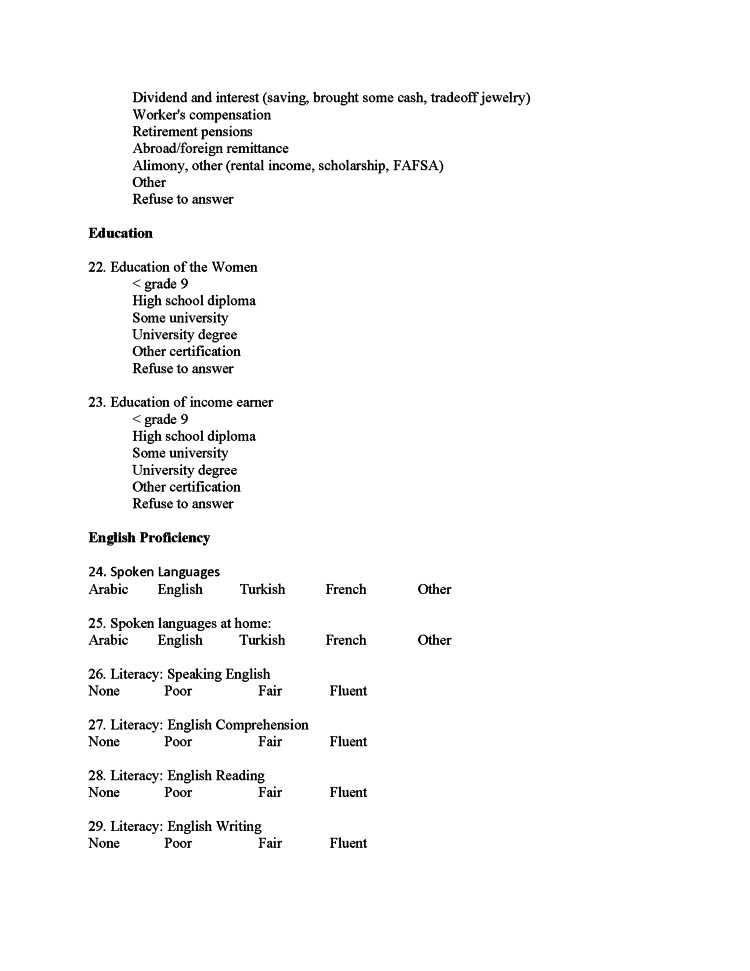
The Comprehensive Questionnaire (Contd.)

**Figure 7:**
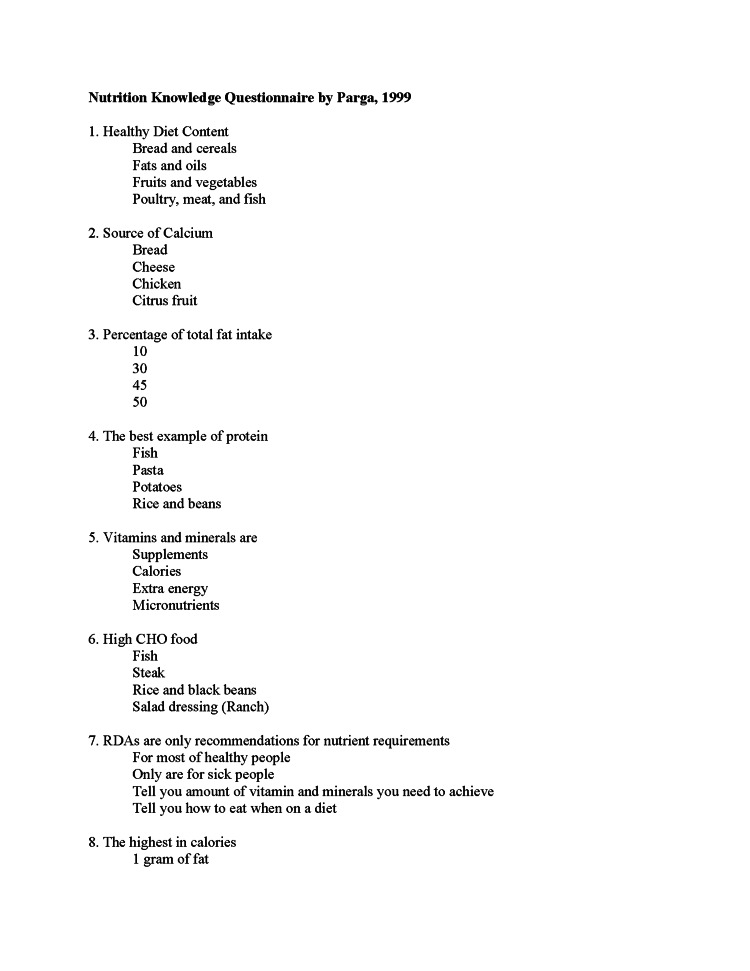
The Comprehensive Questionnaire (Contd.)

**Figure 8:**
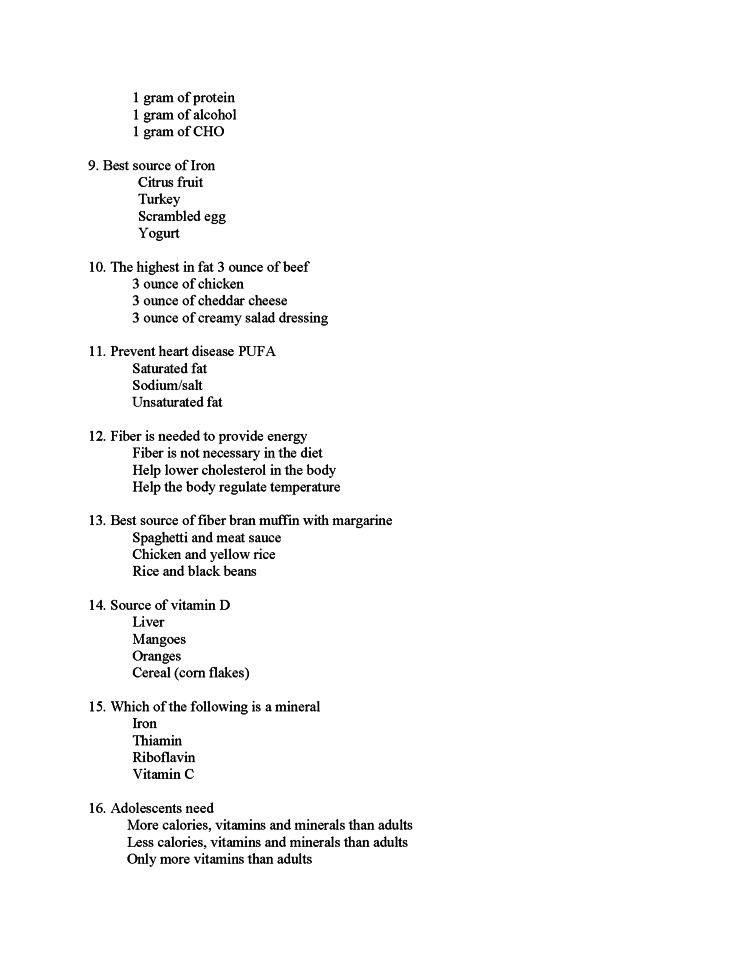
The Comprehensive Questionnaire (Contd.)

**Figure 9:**
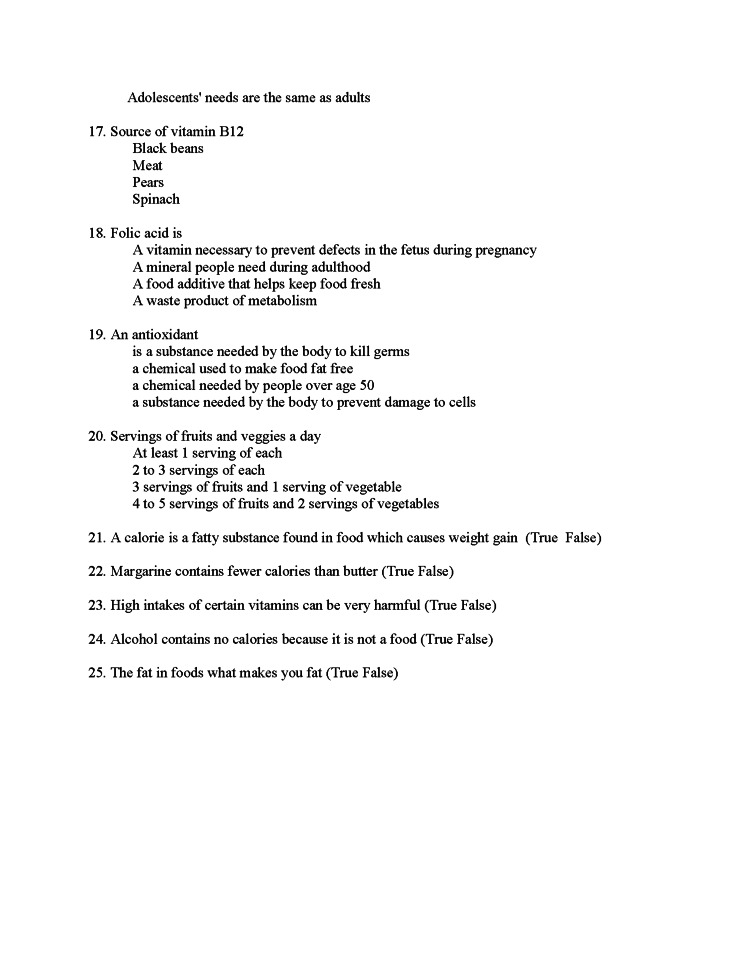
The Comprehensive Questionnaire (Contd.)

## Appendices

Appendix A

Appendix B

Appendix C

**Table 12:** The scoring system of the FSM-USDA model FSM-USDA: Food Security Model-US Department of Agriculture Adapted/reproduced from Bickel et al. [14], with permission

Levels of food insecurity	Assessment questions	System of scale (number of affirmative response)
Food secure	None worried food would run out, food bought did not last	0-2
Food insecure without hunger	Adults not eating balanced meals, child fed low-cost foods, adult cut size or skipped meals, adult eating less than felt they should	3-7
Moderate food insecure with hunger	Adult cut size or skipped meals in 3 or more months in the past 12 months, child not eating enough, adult hungry but did not eat, respondent lost weight, cut size of child’s meals	8-12
Severe food insecure with hunger	Adult did not eat for whole day, child hungry, adult did not eat for a whole day in 3 or more months in the past 12 months, child skipped meal, child skipped meal in 3 or more months in past 12 months, child did not eat for a whole day	13-18

## References

[1] Chang Y, Chatterjee S, Kim J (2014). Household finance and food insecurity. Journal of Family and Economic Issues.

[2] Rose D (1999). Economic determinants and dietary consequences of food insecurity in the United States. J Nutr.

[3] Denney JT, Kimbro RT, Sharp G (2018). Neighborhoods and food insecurity in households with young children: a disadvantage paradox?. Social Problems.

[4] Bartfeld J, Dunifon R (2019). State-Level Predictors of Food Insecurity and Hunger Among Households With Children. https://www.ers.usda.gov/publications/pub-details?pubid=86013.

[5] Nord M, Coleman-Jensen A, Gregory C (2019). Prevalence of U.S. Food Insecurity Is Related to Changes in Unemployment, Inflation, and the Price of Food. https://www.ers.usda.gov/publications/pub-details?pubid=45216.

[6] Bartfeld J, Men F (2017). Food insecurity among households with children: the role of the state economic and policy context. Soc Serv Rev.

[7] Andrews M, Nord M, Bickel G, Carlson S. (2019). Household Food Security in the United States, 1999. https://www.ers.usda.gov/publications/pub-details?pubid=46904.

[8] Coleman-Jensen A, Rabbitt MP, Gregory C, Singh A (2019). Household Food Security in the United States in 2014. United States Department of Agriculture-Economic Research Center.

[9] Nunnery DL, Dharod JM (2017). Potential determinants of food security among refugees in the U.S.: an examination of pre- and post- resettlement factors. Food Secur.

[10] Göttingen GB, Stuttgart GB, Rottenburg LW (2009). Achieving Food and Nutrition Security. Actions to Meet the Global Challenge. A Training Course Reader. https://www.eeas.europa.eu/sites/default/files/2005_achieving_food_and_nutrition_security_inwent.pdf.

[11] Hadley C, Zodhiates A, Sellen DW (2007). Acculturation, economics and food insecurity among refugees resettled in the USA: a case study of West African refugees. Public Health Nutr.

[12] Sleiman D (2018). Innovation: Syrian Women Refugees Use Cooking to Restore Morale and Earn for Their Families. https://www.unhcr.org/news/stories/innovation-syrian-women-refugees-use-cooking-restore-morale-and-earn-their-families.

[13] Sankar R (2019). The impact of socioeconomic factors on food insecurity among Syrian refugees in Florida. https://digitalcommons.fiu.edu/etd/4351/.

[14] Bickel G, Nord M, Price C, Hamilton W, Cook J (2000). Guide to Measuring Household Food Security Revised 2000. Guide to Measuring Household Food Security Revised 2000.

[15] Sankar R, Huffman F (2024). Nutrition knowledge, English adequacy, women's education, and food insecurity among Syrian refugees in Florida. Cureus.

[16] Jensen AC, Nord M, Andrews M, Carlson S (2012). Household Food Security in the United States in 2011. https://cps.ipums.org/cps/resources/food_security/err141.pdf.

[17] Mabli J (2014). SNAP Participation and Urban and Rural Food Security. https://www.google.com/url?sa=t&rct=j&q=&esrc=s&source=web&cd=&ved=2ahUKEwij-cresY-LAxU9k4kEHYQOPMcQFnoECBoQAQ&url=https%3A%2F%2Fwww.mathematica.org%2Fdownload-media%3FMediaItemId%3D%257BDF11CD3B-AAA6-4844-A6C9-BFED551E8AED%257D&usg=AOvVaw1RCGnllPLYZlwtJ5iH1MwE&opi=89978449.

[18] Garvelink WJ, Wedding K (2013). Nutrition and Food Security in the City. https://www.csis.org/analysis/nutrition-and-food-security-city.

[19] Loopstra R, Tarasuk V (2013). Severity of household food insecurity is sensitive to change in household income and employment status among low-income families. J Nutr.

[20] Cafer AM, Kaiser ML (2016). An analysis of difference in predictors of food affordability between rural and urban counties. Journal of Poverty.

[21] Oliveira V (2019). The Food Assistance Landscape: FY 2018 Annual Report. https://ers.usda.gov/sites/default/files/_laserfiche/publications/92896/EIB-207.pdf?v=69266.

[22] Fram MS, Frongillo EA, Jones SJ, Williams RC, Burke MP, DeLoach KP, Blake CE (2011). Children are aware of food insecurity and take responsibility for managing food resources. J Nutr.

